# A Retrospective Sero-Surveillance Study for Antibodies Against Tick-Borne Encephalitis Virus in Norway

**DOI:** 10.3390/v17030291

**Published:** 2025-02-20

**Authors:** Alaka Lamsal, Katrine Mørk Paulsen, Maria Juul Diekmann, Olav Hungnes, Kristian Alfsnes, Else Quist-Paulsen, Daniel Ruzek, Jiri Salat, Petra Strakova, Karen Angeliki Krogfelt, Arnulf Soleng, Rose Vikse, Åshild K. Andreassen

**Affiliations:** 1 Department of Virology, Division for Infection Control and Environmental Health, Norwegian Institute of Public Health, 0456 Oslo, Norway; katrinemorkpaulsen@gmail.com (K.M.P.); diekmann_1993@hotmail.com (M.J.D.); olav.hungnes@fhi.no (O.H.); arnulf.soleng@helsedir.no (A.S.); rose.vikse@fhi.no (R.V.); 2 Department of Natural Sciences and Environmental Health, Faculty of Technology, Natural Sciences and Maritime Sciences, University of South-Eastern Norway, Campus Bø, 3800 Bø, Norway; 3 Department of Bacteriology, Division for Infection Control and Environmental Health, Norwegian Institute of Public Health, 0456 Oslo, Norway; kristian.alfsnes@fhi.no; 4 Department of Microbiology, Oslo University Hospital, 0424 Oslo, Norway; elpa1@ous-hf.no; 5 Department of Experimental Biology, Faculty of Science, Masaryk University, 62500 Brno, Czech Republic; ruzekd@paru.cas.cz; 6 Veterinary Research Institute, 601 77 Brno, Czech Republic; salat@vri.cz (J.S.); petra.strakova@vri.cz (P.S.); 7 Institute of Parasitology, Biology Centre of the Czech Academy of Sciences, 37005 Ceske Budejovice, Czech Republic; 8 Department of Science and Environment, Molecular and Medical Biology, Pandemix Center, University of Roskilde, 4000 Roskilde, Denmark; karenak@ruc.dk

**Keywords:** tick-borne encephalitis virus, TBEV prevalence, Norway, central nervous system infections, seroprevalence study

## Abstract

Tick-borne encephalitis virus (TBEV) is an emerging pathogen that initially causes flu-like symptoms and can progress to central nervous system (CNS) infections. Tick-borne encephalitis (TBE) is an endemic disease in southern coastal counties with regular human cases, while the causative agent, TBEV, is prevalent in ticks in most of the coastal regions of Norway. This study was aimed to understand TBEV infection status across Norway including both TBE endemic and non-endemic areas. For this, we analyzed a total of 1940 residual serum samples from 19 counties of Norway (as of 2016). The samples were initially screened by ELISA, followed by virus neutralization tests for TBEV confirmation. We found a similar TBEV seroprevalence of 1.7% in TBE endemic and 1.6% in non-endemic areas. Since TBE cases are only reported from endemic regions, our findings suggest a potential subclinical or asymptomatic infection and underdiagnosis in non-endemic areas. Notably, only 43% of the ELISA-positive samples were confirmed by virus neutralization tests indicating that not all ELISA positives are true TBEV infections. Additionally, 137 samples of patients presenting with symptoms of CNS infections from a non-endemic area were included. Of these samples, 11 ELISA-positive samples were analyzed for cross-reactivity among flaviviruses. Cross-reactivity was detected with Dengue virus, West Nile Virus, and non-specific reactions. This underscores the importance of using multiple diagnostic tests to confirm TBEV infections. None of the patients with CNS infection was found to be TBE positive, and in the whole cohort, we found a low TBEV seroprevalence of 0.7%.

## 1. Introduction

Tick-borne encephalitis virus (TBEV) is part of the species *Orthoflavivirus encephalitidis*, within the genus *Orthoflavivirus* and the family Flaviviridae. It is primarily transmitted through the bite of an arthropod vector like ticks. It is the causative agent of tick-borne encephalitis (TBE), a potentially severe viral infection affecting the brain and spinal cord [1,2]. Initially presenting with symptoms such as fever, headache, and muscle and joint pain, the infections can progress to more serious conditions, such as meningitis or encephalitis [3]. The virus is geographically widespread, found in regions of Europe and Asia, including Russia and Japan [4,5,6,7]. TBEV is categorized into several subtypes, each with varying implications for disease severity from European, Siberian, Himalayan, Baikalian, and Far-Eastern subtypes [4,5,6,7].

In Scandinavia, particularly Norway and Denmark, human TBEV cases are surprisingly rare despite the widespread presence of the virus in tick populations [8,9,10,11]. This discrepancy raises questions about the potential for subclinical or asymptomatic infections, as suggested by the presence of TBEV antibodies in healthy blood donors [12,13,14,15,16]. Southern counties of Norway, including Buskerud, Vestfold, Telemark, Aust-Agder, and Vest-Agder, are TBE endemic areas from where TBE human cases and the virus prevalence in ticks is regularly reported [17,18]. While the TBEV prevalence in ticks in non-endemic areas in Norway is similar to that in endemic areas, there are no reported human cases from these regions [18,19,20].

The clinical presentation of TBEV infection varies, from non-specific symptoms to encephalitis where many infections may remain unnoticed [21]. When symptoms do manifest, they often appear in two phases, starting with non-specific febrile symptoms and potentially progressing to neurological disorders like meningitis or meningoencephalitis in the second phase [22,23]. Above 70% of the TBE cases caused by TBEV-European are asymptomatic in nature [24]. The primary serological method for TBEV surveillance involves detection of IgG antibodies, which indicate past or recent infection or vaccination. However, the specificity of these tests can be compromised by cross-reactivity with other flaviviruses and often require virus specific neutralization tests for confirmation [25,26,27]. This retrospective study aims to investigate potential TBE infections in areas of Norway where TBEV transmission has not been recognized.

## 2. Materials and Methods

We analyzed a total of 2077 residual serum samples collected from hospital labs and medical doctors. In this, 1940 samples collected in 2016 (1134 males, 806 females) were acquired from the reference biobank for influenza at the Norwegian Institute of Public Health (NIPH). These were residual samples during routine medical examination and blood test, sent by local hospital labs from all the counties of Norway and stored at −20 °C for research purposes. The vaccination status of these samples is unknown. Additionally, 137 serum samples from patients who due to acute onset (<7 days) or worsening of central nervous system (CNS) symptoms (68 males, 69 females) and who were examined by a lumbar Puncture (LP) at Oslo University Hospital (OUS) during 2014–2015 were analyzed [28].

All of these samples were initially screened for antibodies against TBEV with commercial human ELISA, Enzygnost^®^ Anti-TBE/FSME/ETG-virus (IgG) kit and Supplementary reagents for Enzygnost^®^/TMB kit (Siemens Healthcare, GmbH, Marburg, Germany) according to the manufacturer’s protocol, on a DYNEX DS2^TM^ Automated ELISA Processing System (DS2) (Dynex Technologies, Chantilly, VA, USA) at NIPH. IgM analysis was not performed as we were not looking at active TBE infection.

The ELISA-positives were sent for virus neutralization test (VNT) at the Veterinary Research Institute of the Czech Republic. Sera were inactivated by heat (56 °C for 30 min) and diluted 1:4 in Leibowitz L-15 medium (Sigma–Aldrich, Taufkirchen, Germany) supplemented with 3% fetal bovine serum, 100 U/mL penicillin, 100 µg/mL streptomycin, and 1% glutamine (Sigma–Aldrich, Germany). Then, 2-fold serial dilutions of the samples in L-15 medium (50 µL/well) were incubated with 10^3^ PFU/mL of TBEV strain Hypr (50 µL/well) in 96-well plates (TPP, Trasadingen, Switzerland) for 90 min at 37 °C. The virus dose was adjusted to cause an almost confluent cytopathic effect with 90–95% cytolysis. Thereafter, porcine kidney stable (PS) cells were added (3 × 10^4^ cells in 100 µL per well). After five days of incubation, the cytopathic effect was investigated by an inverted microscope CK40 (Olympus, Tokyo, Japan). The highest serum dilution that inhibited the cytopathic effect of the virus was regarded as the endpoint titer. Samples with titer 1:20 and higher were considered positive for the presence of anti-TBEV neutralization antibodies.

The ELISA-positive samples from the cohort of patients with CNS symptoms were tested with Immunofluorescence Flavivirus Profile 2 test from EUROIMMUN (Lübeck, Germany) for cross-reaction with other common reactive flaviviruses like Dengue Virus (DENV), West Nile Virus (WNV), Japanese Encephalitis Virus (JEV), and Yellow Fever Virus (YFV) at NIPH.

Statistical analyses were conducted to assess significant differences in seropositivity rates across defined age groups (0–4, 5–14, 15–24, 25–59, and 60+) and between genders, using SPSS version 28, employing a Chi-squared test of independence with a significance level at α = 0.05

## 3. Results

### 3.1. Seroprevalence of TBEV-Specific Antibodies in Endemic and Non-Endemic Areas

TBEV antibodies were found in 1.6% of samples from all the counties of Norway. The seroprevalence rate was 1.7% in TBEV endemic (7/416) and 1.6% in non-endemic regions (25/1524). Traditional endemic regions like Aust-Agder and Vest-Agder did not show any positives. Among the endemic counties, a higher seroprevalence was noted in Buskerud 6.4% and Vestfold 1.7%. Interestingly, high seroprevalence was found in non-endemic counties like Oslo (4.5%), Rogaland (3.3%), Nord-Trøndelag (3.3%), Troms (2.7%), and Finnmark (2.5%). These findings suggest a broad geographical distribution of TBEV across Norway, including regions not previously identified as concern (Table 1).

#### 3.1.1. Serological Test Specificity

The samples that tested positive for ELISA were subjected to confirmation by neutralization tests (Table 1 and Table 2). Only 43% (33/76) of the ELISA positives were confirmed by VNT. The 12 ELISA positives from patients presenting with CNS symptoms were examined by IFA. One was confirmed positive for TBEV, five were positive for DENV, one for WNV antibodies, and five did not show specific reaction (Table 2).

#### 3.1.2. Gender Distribution

The distribution of TBEV infection across gender showed no statistically significant differences (*p* > 0.05). The seropositivity rate was relatively consistent across all age groups, with no significant difference observed (Chi-squared *p* = 0.93).

## 4. Discussion

**Figure 1:**
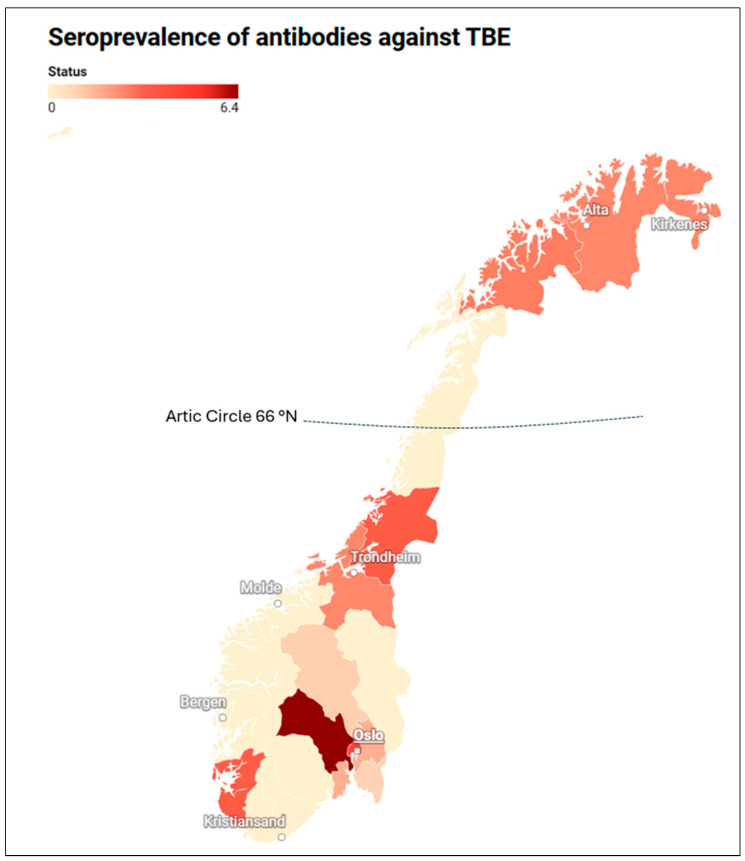
Map of Norway showing seroprevalence (%) of antibodies against tick-borne encephalitis virus in 19 counties of Norway (as of 2016) based on data used in this study.

Tick-borne encephalitis (TBE) is a growing public health concern in Scandinavia [17,29,30]. TBE cases are increasing in Norway, from 87 reported cases between 1994 and 2012 to 283 cases between 2013 and 2022, according to the Norwegian Surveillance System for Communicable Diseases (MSIS). Most of these cases are reported from the southern coastal counties of Agder, Telemark, Vestfold, and Buskerud [17]. The causative agent, TBEV is widely detected in ticks from Østfold County in the southeast to Nordland County in the north, suggesting the potential infection area may be larger than currently understood [8,9,10,11]. We found TBEV seroprevalence in 11 out of 19 counties in Norway, where only five counties were considered risk areas (Figure 1). This may indicate that TBEV infections are more widespread than previously assumed. This study is the first in Norway that examines TBEV seroprevalence in both recognized TBE endemic regions and regions not considered at risk providing insights on the disease distribution in Norway. However, the positive serum samples have an unknown vaccination status and travel history.

We detected TBEV seropositive samples from the counties of Østfold and Akershus from where only two cases are reported. We found seropositive samples in Oslo, Rogaland, Sør-Trøndelag, Nord-Trøndelag, Oppland, Troms, and Finnmark, areas where TBE cases are not reported. This finding is in line with earlier studies that report TBE cases mainly caused by TBEV-EU subtype are likely to be underdiagnosed in Europe and Scandinavia, partly because many infections remain asymptomatic, are misdiagnosed, or are underestimated [15,21,25,31,32]. Studies from Sweden suggest that TBEV infections are likely to be undetected due to asymptomatic infections, misdiagnoses, or underestimation [15,16,22]. Patients with asymptomatic disease or early TBE symptoms that are often comparable to common flu-like illnesses or other CNS-affected diseases may never seek medical assistance or help. Thus, they escape the detection of infection and cause under-diagnosis [16,33]. TBEV underdiagnosis may be a public health issue, and this could include delayed treatment and insufficient resource allocation for disease control. TBE outbreaks in non-endemic areas of France in 2018 and 2020 underscore the importance of TBE surveillance for possible infections beyond endemic areas [34].

In this study, the origin of TBEV infections, whether acquired locally or through travel to endemic regions is unclear. Our tests could not distinguish between infected and vaccinated cases, so some positive cases may be due to vaccination or due to visit to endemic areas within or outside Norway. The southern coastal regions of Norway are popular holiday destinations and TBE vaccination is advised for those frequently exposed to ticks [17]. Approximately 33% to 75% of patients with Lyme disease cannot recall having been bitten by a tick highlighting the difficulty in pinpointing the exact source of these infections [35].

Interestingly, we did not find any seropositive cases from traditionally endemic Aust-Agder and Vest-Agder counties. Since 2017, a majority of TBE cases have been reported from coastal regions of Telemark and Vestfold in the southeast, diverging from the traditionally endemic area in Agder County [17]. This shift could be attributed to an expansion of the endemic area from southernmost coast to southeastern coast or increased awareness and reporting of TBE cases. The disease pattern may be evolving, potentially influenced by factors such as climate change, migration, or alterations in the vector population, leading to a reduction in cases in areas traditionally considered endemic [36,37,38,39,40]. Thortveit et al. [14] found TBE ELISA seropositive in 3.1% of blood donors from Søgne municipality from Vest-Agder County, indicating considerable seroprevalence of TBEV in the area; however, these samples were not confirmed by neutralization tests.

A TBEV seroprevalence of 0.7% was found in patients presenting with symptoms, suggestive of CNS infection. The one TBE-positive patient who had no clinical sign of TBE infection and the seropositivity was considered an incidental finding and unrelated to the symptoms leading to the LP. The blood samples were taken from patients who underwent lumbar puncture due to acute symptoms suggestive of CNS infection. However, most were later diagnosed with other conditions. A retrospective study from Japan found that several patients with neurological disorders had evidence of undiagnosed TBEV infections, suggesting that TBEV circulation may go unrecognized in certain regions [41]. Notably, the TBEV subtype circulating in Japan differs from that in Scandinavia. Our finding is similar to a recent Swedish study, which also reported a low TBEV sero-prevalence in CNS patients and in both Sweden and Norway, the predominant circulating subtype is the European subtype [33]. Nevertheless, our findings based on residual serum samples from hospital labs suggest that even in non- endemic regions, TBE should be considered a potential cause of CNS infection.

TBE has been observed to occur more frequently in men than in women [1,22]. While greater exposure to ticks in males is sometimes cited as a potential cause, the reasons for this sex imbalance is not understood [32]. We did not see distinct gender distribution in our study as reported earlier [33].

Testing of TBEV antibodies can exhibit cross-reactivity among flaviviruses due to shared antigenic similarities, leading to false-positive results [2,42,43,44]. In our study only 44% (33/75) of the ELISA positives could be confirmed with neutralization test, indicating non-specific reactivity or cross-reaction to other flavivirus infections or vaccines (Table 1 and Table 2) [15,25,26]. An ELISA-positive sample that was negative in the neutralization test may suggest the presence of flavivirus-reactive antibodies that may not be specific to TBEV, indicating exposure to other closely related flaviviruses [23,24,39,40]. This suggests that ELISA, while a useful screening tool, may have a significant rate of false positives when used alone for the diagnosis of TBEV.

We tested the 11 ELISA reactive samples from CNS patients with IFA and found antibodies to other cross-reacting viruses like Dengue virus and West Nile virus, as well as unspecific antibodies in some samples. This could be due to pre-existing immunity from previous vaccinations or infections by antigenically similar flaviviruses [2,26,42]. Flaviviruses share conserved epitope-specific parts of the viral protein that are recognized by antibodies that can contribute to cross-reactivity [26]. IFA is often combined with other tests, such as VNT, to identify cross-reactivity in viral serological studies. False-positive ELISA results for West Nile Virus in horses have been linked to cross-reactivity with TBEV antibodies [45]. This underscores the importance of using a combination of serological techniques to enhance the reliability of the test results [26].

Our study suggests a widespread occurrence of TBE in Norway that is consistent with the expanding distribution of TBE in northern Europe and the tick distribution [10,21]. However, this analysis is based on archived samples, and the dynamic nature of vector-borne diseases—affected by climate change, human activities, and shifts in host species distribution—could have modified the current scenario [35,36]. Furthermore, the limitation of having fewer samples tested for cross-reactivity is a potential limitation. Ongoing surveillance and updated research are important to monitor the epidemiology of TBE and improve diagnostic practices.

## 5. Conclusions

This study reports a possible wider distribution of TBEV infections in Norway than currently anticipated. Future research should focus on developing methods to distinguish between infected and vaccinated individuals, as well as conducting retrospective epidemiological studies on TBEV in patients with unexplained neurological symptoms.

## Figures and Tables

**Table 1:** Seroprevalence of TBEV IgG antibodies across Norway *.

County	Number of Samples Analyzed (T)	Number of ELISA Positives	Number of ELISA Positives Confirmed with VNT (P)	Seroprevalence of TBEV % (N = P/T × 100)
Endemic				
Buskerud	78	6	5	6.4
Vestfold	111	4	2	1.7
Telemark	26	0	0	
Aust-Agder	24	0	0	
Vest-Agder	177	0	0	
Overall endemic	416	10	7	1.7
Non-Endemic				
Østfold	112	1	1	0.9
Akershus	119	4	2	1.7
Oslo	134	13	6	4.5
Hedmark	105	2	0	
Oppland	105	4	1	1
Rogaland	121	8	4	3.3
Hordaland	94	1	0	
Sogn og Fjordane	115	1	0	
Møre og Romsdal	118	0	0	
Sør-Trøndelag	80	4	2	2.5
Nord-Trøndelag	123	5	4	3.3
Nordland	107	0	0	
Troms	73	3	2	2.7
Finnmark	118	8	3	2.5
Overall non-endemic	1524	54	25	1.6
Total	1940	64	32	1.6

* The geographic distribution is according to the county borders as in 2016.

**Table 2:** Results from ELISA, VNT, and pan-Flavi IFA tests on TBEV infection confirmation.

Sample Number	ELISA Test	TBEV Confirmatory Test	
		VNT (Titre)	Flavivirus-Specific Antibodies Detected by IFA
1	Positive	Negative (0)	Dengue Virus
2	Positive	Negative (0)	-
3	Positive	Negative (10)	Dengue Virus
4	Positive	Negative (10)	-
5	Positive	Negative (10)	Dengue Virus
6	Positive	Negative (0)	-
7	Positive	Negative (0)	Dengue Virus
8	Positive	Negative (0)	West Nile Virus
9	Positive	Negative (0)	-
10	Positive	Positive (80)	TBE Virus
11	Positive	Negative (10)	-
12	Positive	Negative (10)	Dengue Virus

A sample is considered positive in the VNT when the titer value is ≥20.

## Data Availability

The data supporting the findings of this study are unavailable for public access due to privacy and ethical restrictions on human subject information.
